# Decrease of virulence for BALB/c mice produced by continuous subculturing *of Nocardia brasiliensis*

**DOI:** 10.1186/1471-2334-11-290

**Published:** 2011-10-26

**Authors:** Janeth A Almaguer-Chávez, Oliverio Welsh, Hector G Lozano-Garza, Salvador Said-Fernández, Víktor J Romero-Díaz, Jorge Ocampo-Candiani, Lucio Vera-Cabrera

**Affiliations:** 1 Servicio de Dermatología, Hospital Universitario "José E. González", Monterrey, N.L., 64460 México; 2 Centro de Investigación Biomédica del Noreste, IMSS, Monterrey, N.L., 64720, México; 3 Departamento de Histología, Facultad de Medicina, UANL., Monterrey, N.L., 64460 México

**Keywords:** Mycetoma, Nocardia, attenuation

## Abstract

**Background:**

Subculturing has been extensively used to attenuate human pathogens. In this work we studied the effect of continuous subculturing of *Nocardia brasiliensis *HUJEG-1 on virulence in a murine model.

**Methods:**

*Nocardia brasiliensis *HUJEG-1 was subcultured up to 130 times on brain heart infusion over four years. BALB/c mice were inoculated in the right foot pad with the bacteria subcultured 0, 40, 80, 100 and 130 times (T_0_, T_40_, T_80 _T_100 _and T_130_). The induction of resistance was tested by using T_130 _to inoculate a group of mice followed by challenge with T0 12 weeks later. Biopsies were taken from the newly infected foot-pad and immunostained with antibodies against CD4, CD8 and CD14 in order to analyze the in situ immunological changes.

**Results:**

When using T_40_, T_80 _T_100 _and T_130 _as inoculums we observed lesions in 10, 5, 0 and 0 percent of the animals, respectively, at the end of 12 weeks. In contrast, their controls produced mycetoma in 80, 80, 70 and 60% of the inoculated animals. When studying the protection of T_130_, we observed a partial resistance to the infection. Immunostaining revealed an intense CD4+ lymphocytic and macrophage infiltrate in healing lesions.

**Conclusions:**

After 130 in vitro passages of *N. brasiliensis *HUJEG-1 a severe decrease in its virulence was observed. Immunization of BALB/c mice, with these attenuated cells, produced a state of partial resistance to infection with the non-subcultured isolate.

## Background

Mycetoma in Mexico is mainly caused by actinomycetes with *Nocardia brasiliensis *being predominantly isolated (86.6% of the cases) together with *Actinomadura madurae *(9.6% of the cases)[1]. In the state of Nuevo León, in northeast Mexico, where Monterrey is located, *N. brasiliensis *is isolated in 96% of cases [1]. *Nocardia brasiliensis *is a normal inhabitant of soil and from there it is inoculated by minor trauma with thorns or wood splinters. In humans, the disease is characterized by the production of extensive microabscesses surrounded by granulomatous tissue production. Its pathogenic mechanisms include the production of several proteases that might be involved in tissue destruction [2,3]. The production of an immune cellular response seems to be essential in order to stop infection [4]. However, it has also been claimed that the humoral immune response, particularly IgM subtype antibodies, plays an important role in resistance to infection [5].

Subculturing has been extensively used to attenuate human pathogens. The clearest example is the bacillus of Calmette and Guérin (BCG) which is a *Mycobacterium bovis *attenuated by continuous subculturing on potato-bile medium [6]. Avirulent BCG was produced after 230 serial passages of *M. bovis *and it is utilized to prevent extensive forms of tuberculosis. The mechanisms involved in its avirulence were obscure until recently when by sequencing the complete BCG chromosome, deletions of DNA stretches of up to 10-kb (designated as RD, regions of difference) were observed [7]. The reconstitution of some of these genes restores most of the virulence of *M. bovis *BCG [8].

In the present work we studied if changes in virulence of *Nocardia brasiliensis *HUJEG-1 occurred by subculturing this microorganism 130 times. We also studied the ability of the non-virulent *N. brasiliensis *to induce resistance to the infection with the non-subcultured microorganism.

## Methods

### Subculture method

*N. brasiliensis *HUJEG-1 was used for these experiments; it has been utilized in previous assays [5,9]. Bacterial cultures obtained from mouse lesions were kept frozen at -70°C in 20% skim milk. From these stocks, bacteria were grown on Sabouraud agar at 30°C for 4 to 7 days; then a single colony was placed in a 7 mL sterile Eveljham-Potter device. We added 2.5 mL of sterile saline and the bacterial mass was ground to obtain a homogeneous suspension and turbidity adjusted to McFarland's tube No 1. With this suspension (0.1 mL) we inoculated a 125 ml Erlenmeyer flask containing 33 ml of previously sterilized liquid medium Brain Heart Infusion (BHI). It was incubated with constant agitation at 110 rpm, at 37°C. After 72 h the bacterial mass was harvested by centrifugation at 2,500 rpm for 3 minutes, washed, and ground as above. A new Erlenmeyer flask was inoculated with 0.1 ml of this suspension. These steps were repeated until reaching 130 subcultures (T_130_). Samples were taken every 10 passages and kept frozen at -70°C, including T0. The entire process took about four years.

### Experimental mycetoma in a murine model

Cultures were obtained from the aliquots stored in the deep freezer of passages 40, 80, 100 and 130 (T_40_, T_80_, T_100 _and T_130 _respectively), as well as T0. The inoculums were prepared using a previously published technique [9], and adjusted to 20 mg (wet weight) of *Nocardia brasiliensis *in 50 μL of saline solution. Female BALB/c mice, 8-12 weeks-old were injected with 50 μl of the nocardial suspension in the right footpad and the development of lesions was scored from 0 (for no inflammatory changes) to 4+ (extension of the lesions beyond the ankle of the animal with extensive production of inflammation and abscesses) as previously described [9]. The thickness of each lesion was measured with a caliper every week for 12 weeks. The study was approved by the Comité Local de Investigación en Salud No. 1908, Centro de Investigación Biomédica del Noreste, IMSS, and the animal handling was done according to our institutions' guidelines.

### Induction of infection resistance in a murine model

In order to study if infection with subcultured *N. brasiliensis *produced a state of immune resistance we inoculated a group of animals with *N. brasiliensis *subcultured 130 times in the right foot pad. After 12 weeks the left footpad was inoculated with the non-subcultured bacteria (T0). As a control we inoculated a group of animals of the same age with the non-subcultured isolate in the right footpad. In all cases the development of lesions was scored and measured as described above.

### Histopathological analysis comparing T_0 _and T_130 _strains

For histopathological evaluation of the infection process we obtained biopsies of the inoculated footpad on weeks 1, 3, 5, 7, 9 and 12 post-inoculation in both groups: the T_130 _re-inoculated group and the control. Biopsies were stored in 4% formalin for further processing and staining with H&E, Kinyoun, and PAS. To identify the subsets of the inflammatory cells, the tissue samples were stained with antibodies against CD4 (helper T cells), CD8 (suppressor/cytotoxic T cells) and CD14 (monocytes). Briefly, sections from affected feet destined for immunohistochemistry were deparaffined, rehydrated and subjected to a sequence of incubation steps starting with sodium citrate (0.01 M) for epitope recuperation. After blocking endogenous peroxidase activity with 1% hydrogen peroxide in methanol, sections were incubated in a humidity chamber during 18 h at 4°C with polyclonal anti-CD4, anti-CD8 and anti-CD14 (Dako Corp., Carpinteria, CA) diluted 1:200 in PBS. Following rinses in PBS, sections were incubated for 20 min at room temperature with biotinylated goat anti-rabbit antibody, and diluted 1:500 in PBS. This was followed by rinses in PBS and 20 min humidity chamber incubation in streptavidin-biotin. Peroxidase activity was visualized by incubating the sections with 3,3',-diaminobenzidine and counterstaining with hematoxilin.

### Statistical analysis

The statistical analysis was performed with Statgraphics under the heading of The StatAdvisor and also with SPSS, Sigma Plot and Excel 2007. For comparison between groups an ANOVA analysis was conducted and as an alternative a Kruskal-Wallis test was applied. A *P < 0.05 *was considered statistically significant.

## Results

### I. Effect of subculture on *Nocardia brasiliensis *HUJEG-1 virulence

**Figure 1:**
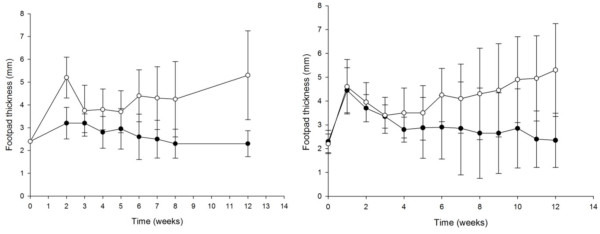
**Development of lesions of mice infected with T_40_, and T_80_**. Mice were infected with 20 mg (wet weight) of bacterial fragments from T_40 _(left) and T_80 _(right). The footpad thickness was measured weekly; each point represents the mean of 20 animals and bars represent the standard deviation. As a control we inoculated a group of animals with T0 (open circles). When analyzed with the ANOVA test, a significantly difference was observed with regard to the control at 12 weeks post inoculation (***P ***< 0.001) in both cases.

In Figure 1 we show the evolution of the natural infection in mice when inoculating the bacteria subcultured for 40 and 80 passages compared with the control (T_0_). In the animals inoculated with T0 there is an initial inflammation due to an intense antigenic stimulation produced by the inoculum, followed by a decrease in the thickness of the footpad. Mycetomas appear after 5-6 weeks after infection. Twelve weeks after inoculation there is an intense increase in the footpad size characterized by the presence of abscesses and fistulae.

When inoculated with the subcultured *N. brasiliensis *strains a different behavior was observed. After the initial inflammatory response the mean thickness of the footpad decreased significantly after five weeks post-inoculation in all cases. The formation of mycetoma lesions (more than +) at week 12, was observed in 10, 5, 0 and 0 percent of the animals inoculated with T_40_, T_80_, T_100 _and T_130 _respectively (Table 1). Their controls produced mycetoma in 80, 80, 70 and 60% of the inoculated animals. At week 12, the development of lesions in the groups inoculated with the subcultured isolate was compared with its own control (inoculated with T0), and analyzed with the ANOVA test. A *P *< 0.001 was observed in all cases.

**Table 1:** Production of mycetoma lesions in BALB/c mice infected with the subcultured *N.brasiliensis *HUJEG-1 isolate

Inoculum utilized				
	
Development of lesions	T40	T80	T100	T130
0+	18/20	18/20	20/20	28/30
1+	0/20	0/20	0/20	0/30
2+	2/20	0/20	0/20	0/30
3+	0/20	1/20	0/20	0/30
4+	0/20	0/20	0/20	0/30

### II. Induction of infection resistance in a murine model

**Figure 2:**
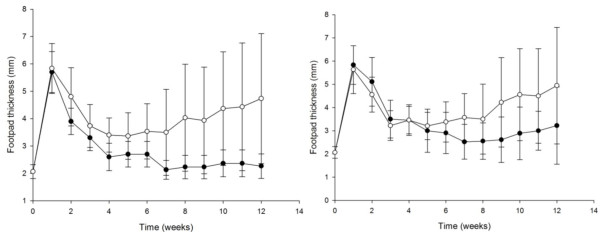
**Evolution of mycetoma lesions in animals inoculated with T_130 _(left) and re-inoculated with the T0 (right)**. Each point represents the mean of 30 animals; bars represent the standard deviation. An equal group of animals was inoculated with T0 (open circles). At twelve weeks after infection statistically significant differences were observed in both cases (***P ***< 0.001 and ***P ***= 0.021).

The animals inoculated with *N. brasiliensis *subcultured 130 times produced no lesions until 12 weeks after inoculation (*P *< 0.001). At the end of 12 weeks, these animals were challenged with the non-subcultured isolate in the left footpad (Figure 2). The natural evolution was similar in the first five weeks after inoculation with the non-subcultured isolate, but subsequently, we observed a decrease in the development of lesions as compared to their respective controls (*P *= 0.021) (*P *value determined by a variance test ANOVA).

### III. Histological study of the lesions

**Figure 3:**
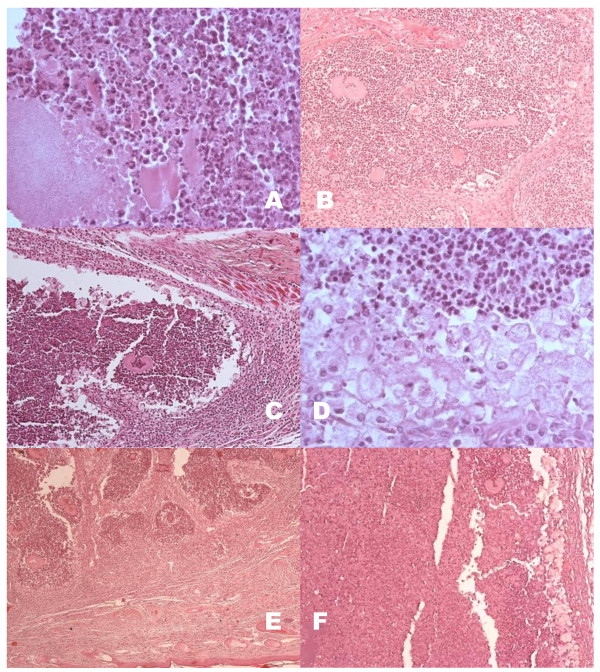
**Histological features of BALB/c mice inoculated with *N. brasiliensis *HUJEG-1 without subculturing**. A: After one week fragments of the colonies are seen surrounded by PMNs; 400×. B: At three weeks we can observe well formed grains; 100×. C: Five weeks after infection well formed grains in micro-abscesses (100×) surrounded by a layer of foamy cells are observed (D; 600×). E: At seven weeks there is an abundance of grains present in the lesions arranged in a multilocular form; 100×. F: Biopsy taken after 12 weeks showing grains and giant foamy cells immersed in huge abscesses of necrotic PMNs; 100×. H&E stain.

In mice inoculated with the non-subcultured *N. brasiliensis *we observed abundant polymorphonuclear cells (PMN) surrounding the *N. brasiliensis *fragments inoculated (not yet constituted in grains) after 1 week of inoculation (Figure 3). Two weeks later, grains were observed surrounded by PMN and fibrosis was visible while at the periphery a layer made up of abundant foamy macrophages was evident. Seven weeks post-inoculation abundant grains and microabscesses contained in concentric fibrotic walls were observed. And at the 12 weeks of inoculation gigantic foamy cells were present around the abscesses with grains floating in a huge abscess composed of PMN and necrotic cells. Macroscopically it was assigned as a 4+ lesion.

**Figure 4:**
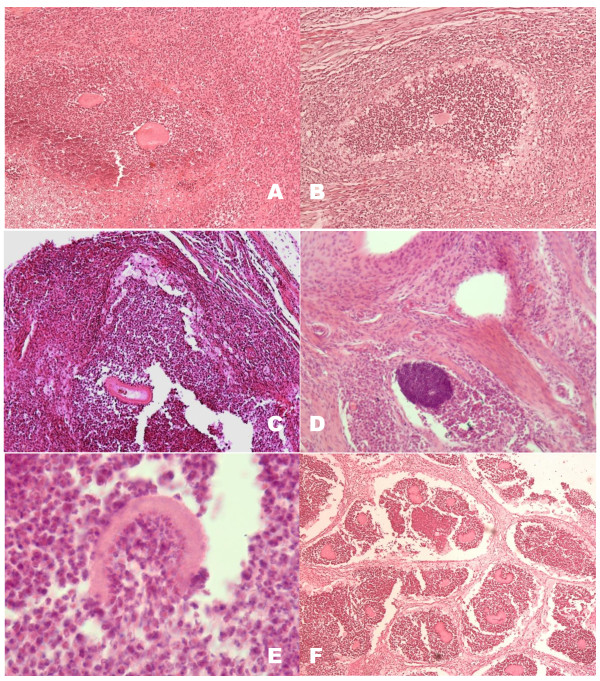
**Histological features of BALB/c mice inoculated with *N. brasiliensis *HUJEG-1 T130 and re-inoculated with T0**. A and B: In the first 3 weeks findings are similar to those of the animals inoculated with T_0_; 100×. C, and D: At five weeks an intense mononuclear infiltrate was observed (100×), with thin walled grains and abundant PMNs attached to them (E; 600×). F: At twelve weeks, some animals presented lesions with grains in microabscesses surrounded by fibrotic walls; 100×. H&E stain.

The animals inoculated with T_130 _and re-inoculated with T0 showed an initial PMN infiltrate around the inoculum's fragment (Figure 4). Three weeks post-infection, micro abscesses containing the grains or inoculum fragments surrounded by a layer of foamy macrophages were present. At week five, microabcessess were formed with smaller amounts of foamy cells than in those in animals inoculated with T_0 _and grains in different stages of destruction were observed. A strong mononuclear infiltrate in the periphery of the microabscesses amongst the collagen fibers was evident. After five weeks, there was a decrease in the number of animals with lesions (Figure 2). Biopsies taken from animals presenting lesions at 12 weeks showed abundant grains in microabscesses encapsulated in fibrotic walls.

### IV. Immunostaining of the lesions

In Table 2, the results of the immunostained biopsies are shown. In the animals inoculated with the non-subcultured isolate, we observed few CD8+ T lymphocytes, mostly situated around microabscesses, among the fibrosis; macrophages (CD14+ cells) were rarely seen. CD4+ T lymphocytes were observed more numerous. In heavily infected mice (12 weeks after infection), there were few lymphocytes and macrophages, perhaps due to the fact that most of the lesions were composed of abscesses.

**Table 2:** Results of immunostaining biopsies of lesions with antibodies against CD4, CD8 and CD14.

**Weeks after Reaction to Inoculation antibody against:**						
	
	CD4		CD8		CD14	
	
	T_0_	T_130_	T_0_	T_130_	T_0_	T_130_
1	(-)	±	(-)	(-)	(-)	(-)
3	++	(-)	+	(±)	(±)	(±)
5	++	+++	+	(±)	+	++++
12	±	++	±	(±)	(±)	+++

**Figure 5:**
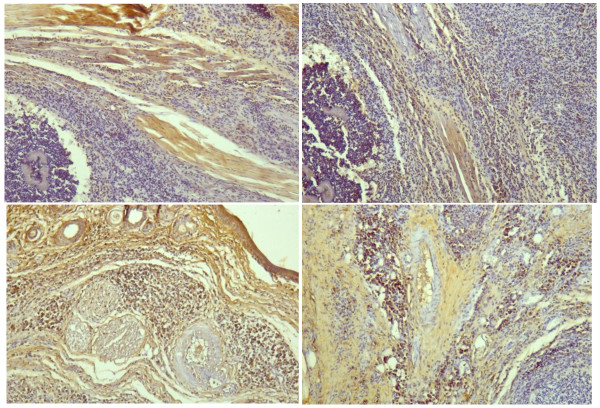
**Immunostaining of biopsies of lesions at five weeks postinfection**. Top: Mouse inoculated with the non-subcultured isolate showing positive cells for anti-CD4 (left) and for anti-CD14 (right); 100×. Bottom: Foot-pad biopsy of mouse inoculated with T_130_. CD4+ (left) and CD14+ (right) T-cell lymphocytes are seen abundantly surrounding the microabscesses among the scarring tissue; (100×).

In the animals inoculated with T_130 _and then challenged with the non-subcultured bacteria a different pattern emerged. CD8+T lymphocytes were rarely seen (Table 2) in all samples analyzed, while CD4+ lymphocytes were much more numerous after 5 weeks of inoculation (Figure 5). At this time clinical lesions decrease in size and most animals started to heal. Macrophages (CD14+ cells) were observed very abundantly in clusters from the fifth week in the scarred areas of the lesions, constituting most of the cells present (Figure 5).

## Discussion

Five weeks after infection the lesions started to heal in the animals inoculated with T_130 _and re-inoculated with T0. At this time the lesions were microscopically characterized by the presence of hollow and semi-destroyed grains as well as by the presence of an intense mononuclear infiltrate. These microscopical features are similar to those observed in Lewis rats infected with *N. brasiliensis *[10]. These animals are naturally resistant to the infection even when using high amounts of bacteria. They present an initial inflammatory reaction, but from this point the lesions start to heal spontaneously; in the microscope, thin-walled, semi-destroyed grains are observed. This suggests that immunization with the attenuated cells (T_130_) can induce an immune state of resistance, although it is not complete. It will be important to assay re-immunizations with the whole bacteria or crude extracts to determine if the level of resistance can be increased.

The presence of small amounts of lymphocytes and macrophages in biopsy tissue of human cases of actinomycetoma has been reported [11,12]. They are present in low numbers and are situated at the periphery of the microabscesses; similar findings were observed in this work in mice infected with T_0_. In leprosy, two patterns of in situ localization of T lymphocytes subsets have been described: in tuberculoid lesions CD8+ (OKT8+) cells are found around the granulomata, and CD4+ (OKT4+) cells are distributed throughout the granuloma [13]. In lepromatous patients both subsets are distributed throughout the granuloma with no predilection for the periphery. The organization of T lymphocyte subsets in tuberculoid leprosy reflects an effective immune response. In actinomycetoma, there are not true granulomas, instead the microorganisms are contained inside microabscesses surrounded by a layer of foam laden macrophages. These microscopic features have been described before as "*N. brasiliensis *lesions" which are different of those presented by "*N. asteroides*" [14]. In the case of mice immunized with T_130 _and then challenged with T0 we observed an increase in CD4+ lymphocytes as well as an abundant infiltrate of macrophages (CD14+ cells) five weeks after immunization. Mononuclear cells were always located in clusters outside the microabscesses in the scar tissue. It appears that this remarkable increase in the number of macrophages is responsible for the elimination of *Nocardia *and consequent healing of the lesions in our experiments. The identification of the antigens of *N. brasiliensis *which elicits this cellular immune response, as well as the immune mechanisms (cytokines and cells) involved in the destruction of the grains, will help to provide a better understanding of the resistance mechanisms to *N. brasiliensis*.

Studies on bacterial evolution have permitted the analysis of changes in the genome produced by adaptation to a particular medium or niche. In *Escherichia coli *it has been observed that mutations, deletions, IS insertions, duplications and inversions in the chromosome appear in as short as in 2000 generations [15] and gene changes increase in a linear form until 20,000 generations producing the loss of about 1.2% of the original chromosome (during a total of 20 years of subculturing). Although it is a large amount of deleted, DNA most of the mutations observed were synonymous (without sense), not affecting the biological properties of the bacterium. In our case it seems that *Nocardia *needs only a few generations to produce changes in its virulence, since after 40 subcultures we observed a remarkable difference in virulence. In *Burkholderia cenocepacia *as little as 500 generations made this bacterium unable to kill the nematode *Caenorhabditis elegans *[16]. In that work 1000 generations took about 152 daily subcultures. Given the longer generation time of Nocardia it took about 4 years to achieve 130 subcultures. It is difficult to calculate the exact number of generations since *Nocardia brasiliensis *divides by producing filaments rather than by binary fission. It will be necessary to design an experiment to overcome this problem and determine the number of generations in order to compare our results with other experiments in microbial evolution.

In the case of *M. bovis*, a phylogenetically related organism, it took about 230 subcultures in potato-ox bile liquid medium to decrease its virulence for several mammals, including calves, horses, and man [6]. BCG attenuation has been explained by the loss of large DNA stretches (known as the regions of difference, RD) [7], with the most important being RD1 which includes ORF's coding for proteins ESAT-6 and CFP10. In the case of *N. brasiliensis*, we observed a significant decrease in virulence, however since we do not yet have the *N. brasiliensis *genome sequence, we can not determine the genetic changes responsible for the decrease in virulence. DNA sequence analysis of the complete genome of *N. brasiliensis *will allow us to determine the genes involved in *Nocardia brasiliensis *virulence.

## Conclusion

In conclusion, we observed a remarkable decrease in the virulence *of Nocardia brasiliensis *HUJEG-1 after subculturing it 130 times in vitro, compared to the parent strain. This can be an excellent model to study the immunological mechanisms involved in resistance in mycetoma, since a previous inoculation with the attenuated *N. brasiliensis *provides protection against the infection with the parent strain or T0.

## Competing interests

The authors declare that they have no competing interests.

## Authors' contributions

LV-C and JAA-Ch. were responsible for conception and design of the study, conduct of analysis, interpretation of data, and drafting and revision of the manuscript; JO-C and OW were involved in design of the study, conduct of analysis, interpretation of data and critical revision of the manuscript; HG-L and SS in design of the study, interpretation of data, and critical revision of the manuscript. VJR-D helped with the immunostaining techniques. All authors read and approved the final manuscript

## Pre-publication history

The pre-publication history for this paper can be accessed here:

http://www.biomedcentral.com/1471-2334/11/290/prepub
